# Supplementary material for: EnzML: multi-label prediction of enzyme classes using InterPro signatures
Source: BMC Bioinformatics. 2012 Apr 25;13:61. doi: 10.1186/1471-2105-13-61 (PMC3483700; doi:10.1186/1471-2105-13-61)
Supplement: Addtional file 5 — The Java code to format the data files, evaluate and predict. The file enzml_java_code.tar.gz contains the Java code used to format database data to ARFF and XML formats, to execute cross and train-test (jackknife) evaluations and to record evaluation results to database. More information is included in the readme.txt file and the Javadoc files. The code can be used with a MySQL database. To use a different database software, other JDBC drivers might be required. [file 1471-2105-13-61-S5.gz › java_code/enzml2011/doc/uk/ac/ed/inf/enzml/mulan/learn/traintest/package-frame.html]

uk.ac.ed.inf.enzml.mulan.learn.traintest


uk.ac.ed.inf.enzml.mulan.learn.traintest

Classes 
  
MulanTrain
  
TrainTestEvaluator
  
TrainTestEvaluatorSerialised
  
TrainTestExperimenter
  
TrainTestExperimenterSerialized
  
TrainTestFullRun
